# Using inflammatory parameters for safe and early discharge after minimally invasive colorectal surgery for colorectal cancer

**DOI:** 10.1007/s10151-025-03134-2

**Published:** 2025-04-07

**Authors:** B. D. N. Dos Santos, C. Beruti, J. Azevedo, I. Herrando, P. Vieira, H. Domingos, R. Heald, L. Fernandez, A. Parvaiz

**Affiliations:** 1 Digestive Unit, Champalimaud Foundation, Av Brasilia, 1400-038 Lisbon, Portugal; 2 Hospital Universitario Austral, Buenos Aires, Argentina; 3 Faculty of Medicine, University of Lisbon, Lisbon, Portugal

**Keywords:** Inflammatory markers, Colorectal cancer, Early discharge, Minimally invasive surgery

## Abstract

**Background:**

Minimally invasive surgery has become the gold standard for colorectal cancer treatment. Approximately 40% of patients undergoing elective colorectal resection develop postoperative complications. The median time to clinical diagnosis of a postoperative complication ranges between 5 and 8 days. Early detection of complications can reduce their morbidity and negative impact. This study aims to evaluate the effectiveness of routine postoperative inflammatory markers in predicting early postoperative complications in patients undergoing elective minimally invasive surgery for colorectal cancer.

**Methods:**

This study was conducted at a single center and is a retrospective analysis of a prospectively mantained database. We included 397 consecutive patients who underwent elective minimally invasive surgery for colorectal cancer between May 2012 and September 2023. Routine inflammatory parameters, including C-reactive protein, Glasgow Prognostic Score, and neutrophil–lymphocyte ratio, were analyzed to identify those associated with postoperative complications. The cutoff values for these markers were determined using receiver-operating characteristic (ROC) curve analysis with the Youden index method.

**Results:**

Of the patients, 29.2% experienced postoperative complications, with major complications (Clavien–Dindo ≥ III) occurring in 11.3%. On postoperative day 3, C-reactive protein level < 125 mg/L, Glasgow Prognostic Score < 2.12, and neutrophil–lymphocyte ratio < 5.26 were significantly associated with lower risk of postoperative complications (*p* < 0.0001). NLR was the best parameter to identify patients unlikely to experience a postoperative complication on day 3, with a cutoff value of 5.26 and a negative predictive value (NPV) of 83%.

**Conclusions:**

Neutrophil–lymphocyte ratio, C-reactive protein, and Glasgow Prognostic Score on POD3 can predict postoperative complications in patients who undergoing minimally invasive surgery for colorectal cancer. These inflammatory markers demonstrated high negative predictive value, effectively identifying patients who are unlikely to develop complications and providing valuable information for safe early discharge.

## Introduction

Minimally invasive surgery (MIS) has become the gold standard for colorectal cancer treatment [1, 2]. Less invasive approaches, such as laparoscopic or robotic surgery, combined with preoperative and postoperative enhanced recovery pathways aimed to reduce postoperative morbidity. However, approximately 40% of patients undergoing elective colorectal resection will develop postoperative complications, the most frequent being surgical-site infection, ileus, bleeding, and anastomotic leakage (AL) [3].

Surgical complications are associated with short-term effects such as increased hospital stay, readmissions, and reoperations, but also with worse long-term oncological outcomes [4]. The median time to clinical diagnosis of a postoperative complication ranges between the 5th and 8th postoperative days [5–8]. A delay in diagnosis may be detrimental to the patient, potentially increasing the risk of adverse outcomes. On the other hand, early detection of postoperative complications plays a crucial role in reducing their negative impact by implementing more effective and timely strategies.

Additionally, early detection of posoperative complications may allow their management with minimally invasive techniques, resulting in faster recovery times.

The use of inflammatory markers (IM) including postoperative C-reactive protein levels, white blood cell count, platelet count, and neutrophil–lymphocyte ratio, among others, has been shown to be particularly useful for the detection of postoperative complications [9–11]. However, most of these studies had heterogeneous inclusion criteria regarding indications for colorectal surgery and surgical approach, including both open and minimally invasive procedures.

The main objective of this study is to evaluate the ability of routine postoperative inflammatory parameters in the early prediction of postoperative complications following elective minimally invasive for colorectal cancer surgery.

## Materials and methods

A single-center retrospective analysis of a prospectively maintained database of all consecutive patients who underwent elective minimally invasive surgery (MIS) for colorectal cancer, between May 2012 and September 2023, was performed. Patients older than 18 years diagnosed with primary colorectal adenocarcinoma, suitable for curative resection during the study period were included.

Patients diagnosed with recurrent CRC, benign lesions, nonadenocarcinoma tumors, metastatic disease, open surgery, or unavailable inflammatory parameters were excluded.

This retrospective study was conducted in accordance with institutional guidelines, which did not require formal ethics committee approval for the use of anonymized data. However, all patients provided written informed consent for the use of their anonymized information in research purposes.

### Clinical and demographic parameters

Patient clinical variables evaluated were age, gender, body mass index (BMI), and American Society of Anesthesiologists (ASA) grading.

The preoperative parameters evaluated were clinical tumor staging, tumor location, and previous treatment. Tumor staging was according to the tumor–nodule–metastasis (TNM) system of the American Joint Committee on Cancer (8th edition) [12]. Tumor location was determined using surgeon’s intraoperative reports or preoperative computed tomography when these were not available.

### Perioperative data

Minimally invasive procedures were performed either laparoscopically or entirely robot-assisted using a da Vinci Xi system (Intuitive Surgical, Sunnyvale, CA, USA).

The nature and severity of all postoperative complications were classified according to Clavien–Dindo classification [13]. Postoperative complications were defined as those that occurred during the first 30 days post-surgery. The presence of a classification equal to or greater than III was considered a major complication (≥ III). The conversion rate and the need for blood transfusion were also registered.

Tumor pathological staging was also based on the TNM system [12]. The data were collected from the anatomopathological report.

### Inflammatory parameters

The inflammatory parameters were measured on postoperative day 1 (POD1), postoperative day 3 (POD3), and postoperative day 5 or the discharge day (POD5), if discharge occurred before day 5. A rise in CRP is expected following the tissue damage caused by the surgical procedure, with a peak typically observed around 48 h. However, the kinetics of other inflammatory markers may differ. In uneventful cases, the levels are expected to decrease on POD3 [8, 14]. For this reason, no measurements were recorded on postoperative day 2.

The inflammatory markers (IM) analyzed were those routinely assessed during standard postoperative management, including: white cell count (WCC), neutrophil count (NC), lymphocyte count (LC), platelet count (PC), albumin (ALB), C-reactive protein (CRP), and procalcitonin (PCT).

Ratios of these parameters were also performed, such as the Glasgow Prognostic Score (GPS), based on the serum elevation of CRP and decrease in ALB concentration, neutrophil–lymphocyte ratio (NLR), platelet–lymphocyte ratio (PLR), and procalcitonin–albumin ratio (PAR).

### Outcomes

The primary outcome evaluated was the ability of inflammatory parameters to predict postoperative complications.

### Statistical analysis

Qualitative variables are presented as frequency and percentage, and quantitative variables as median and standard deviation.

For univariate analysis, categorical variables were analyzed with a chi-squared test and continuous and discrete variables were analyzed using the independent-sample Student *t*-test.

Multivariate analysis was performed for selected variables with *p* < 0.3 in the univariate analysis.

Receiver-operating characteristic (ROC) curves were created at POD1, POD3, and POD5 for select markers (*p* < 0.05) to determine sensitivity, specificity, negative and positive predictive values and accuracy for each inflammatory parameter to identify the optimal value for predicting postoperative complications using the Youden index method.

XLSTAT^®^ (version 2023.3.0) software was used to perform statistical analysis. Statistical significance was set at *p* < 0.05.

## Results

**Fig. 1:**
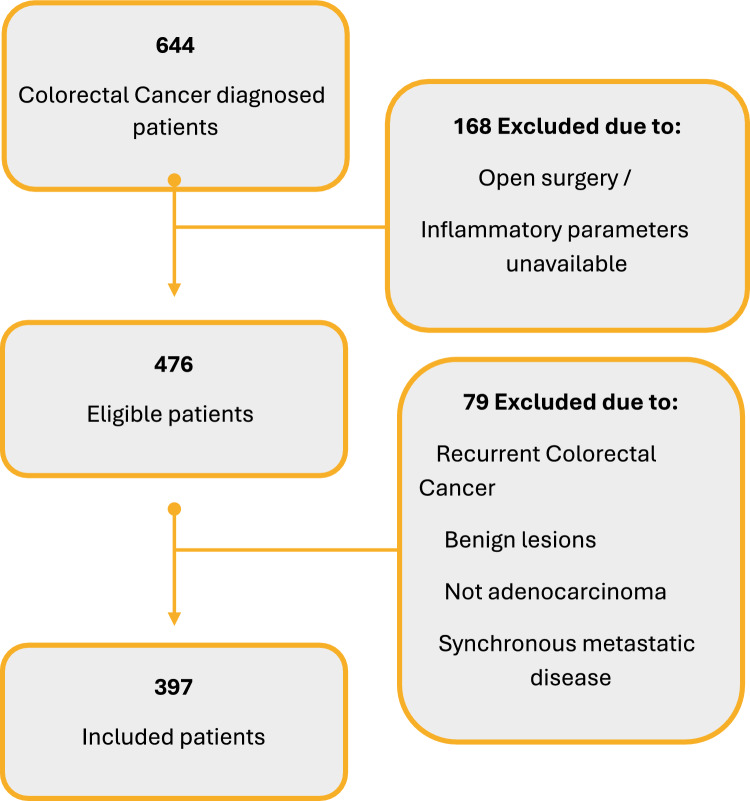
Flowchart of eligibility and exclusion criteria

A total of 644 patients were identified during the study period. After applying exclusion criteria, 397 patients were included in our analysis (Fig. 1).

Of the 397 patients included, 217 (54.7%) were male with a median age of 66.6 years, 216 (54.4%) patients had colon cancer, and 181 (45.6%) had rectal cancer.

Surgeries were performed robotically in the majority of patients (57.4%, *n* = 228) and laparoscopically in 42.6% (*n* = 169). Complete demographic characteristics are presented in Table 1.

**Table 1:** Clinical and demographic characteristics of the studied population and comparison between postoperative complicated and not complicated groups

	Total (*n* = 397)	Without complications (*n* = 281)	With complications (*n* = 116)	Univariate *p*-value	Multivariate OR (*p*-value)
Age	66.6 ± 12.5	66.4 ± 12.8	69.4 ± 11.6	**0.004**	3.4 (**0.001)**
BMI	26.2 ± 4.3	25.7 ± 4.0	27.2 ± 4.7	**0.002**	2.6** (0.010)**
Gender (M/F)	217/180 (54.7%/45.3%)	143/138 (50.9%/49.1%)	74/42 (63.8%/36.2%)	**0.019**	
ASA score				**0.039**	
I	12 (3.0%)	9 (3.2%)	3 (2.6%)		
II	294 (74.1%)	217 (77.2%)	77 (66.4%)		
III	85 (21.4%)	53 (18.9%)	32 (27.6%)		
IV	6 (1.5%)	2 (0.7%)	4 (3.4%)		
Tumor location					
Rectum	181 (45.6%)	113 (40.2%)	68 (58.6%)	**0.002**	
Colon	216 (54.4%)	168 (59.8%)	48 (41.4%)		
Clinical staging, cT					
I or II	85 (29.8%)	59 (30.3%)	26 (28.9%)	0.430	
III or IV	200 (70.3%)	136 (69.7%)	64 (71.1%)		
cN					
0	158 (53.2%)	116 (56.6%)	42 (45.7%)	0.284	
+	139 (46.8%)	89 (43.4%)	50 (54.3%)		
Neoadjuvant therapy	73 (18.4%)	36 (12.8%)	37 (31.9%)	**0.0002**	3.5** (0.001)**
Surgical approach				0.061	
Laparoscopic	169 (42.6%)	128 (45.6%)	41 (35.3%)		
Robotic	228 (57.4%)	153 (54.4%)	75 (64.7%)		
Conversion (n)	9 (2.3%)	6 (2.1%)	3 (2.6%)	0.784	
Blood transfusion	8 (2.0%)	3 (1.1%)	5 (4.3%)	**0.037**	

Bold values indicate statistical significance (*p* <0.05)

### Postoperative complications

Postoperative complications occurred in 116 patients (29.2%). Grade II was the most common (50.0%), and major complications (Clavien Dindo ≥ III) were observed in 11.3% of the entire population (Table 1).

A complete analysis of factors associated with postoperative complications is presented in Table 1.

### Predictive parameters for postoperative complications

Most of the inflammatory parameters analyzed were associated with the development of postoperative complications, as seen in Table 2.

**Table 2:** Inflammatory parameters on POD1, POD3, and POD5

	Total (*n* = 397)	Not complicated (*n* = 281)	Complicated (*n* = 116)	Univariate *p*-value
WCC (× 10^9^/L)				
POD1	10.9 ± 3.1	10.9 ± 3.1	10.7 ± 2.9	0.480
POD3	7.5 ± 2.8	7.0 ± 2.1	8.5 ± 3.7	** < 0.0001**
POD5	6.6 ± 2.6	6.3 ± 1.9	7.2 ± 3.5	**0.004**
NC (× 10^9^/L)				
POD1	8.8 ± 2.9	8.8 ± 3.0	8.8 ± 2.6	0.976
POD3	5.4 ± 2.6	4.8 ± 1.9	6.6 ± 3.4	** < 0.0001**
POD5	4.5 ± 2.5	4.1 ± 1.8	5.3 ± 3.3	** < 0.0001**
LC (× 10^9^/L)				
POD1	1.2 ± 0.6	1.3 ± 0.5	1.1 ± 0.6	**0.001**
POD3	1.3 ± 0.6	1.4 ± 0.6	1.1 ± 0.6	** < 0.0001**
POD5	1.2 ± 0.6	1.4 ± 0.6	1.0 ± 0.6	** < 0.0001**
NLR				
POD1	9.3 ± 6.6	8.8 ± 6.4	10.6 ± 6.8	**0.011**
POD3	5.2 ± 4.5	4.1 ± 3.0	8.0 ± 6.1	** < 0.0001**
POD5	4.8 ± 5.3	3.5 ± 2.3	7.1 ± 7.8	** < 0.0001**
PC (× 10^9^/L)				
POD1	212.3 ± 66.6	212.8 ± 66.6	211.3 ± 66.7	0.842
POD3	211.3 ± 69.5	208.7 ± 67.1	217.7 ± 74.7	0.239
POD5	228.5 ± 72.9	222.7 ± 69.8	239.1 ± 77.6	0.054
PLR				
POD1	216.7 ± 138.8	201.6 ± 118.8	253.2 ± 173.3	**0.001**
POD3	201.8 ± 141.6	170.0 ± 88.3	278.9 ± 203.9	** < 0.0001**
POD5	228.2 ± 141.3	186.7 ± 88.3	304.6 ± 183.2	** < 0.0001**
ALB (g/dL)				
POD1	3.6 ± 0.4	3.6 ± 0.4	3.5 ± 0.4	**0.031**
POD3	3.5 ± 0.4	3.5 ± 0.4	3.4 ± 0.5	**0.025**
POD5	3.4 ± 0.5	3.5 ± 0.5	3.3 ± 0.6	** < 0.0001**
CRP (mg/dL)				
POD1	6.1 ± 3.8	5.7 ± 3.6	6.9 ± 4.0	**0.006**
POD3	9.8 ± 7.2	7.8 ± 5.0	14.8 ± 9.0	** < 0.0001**
POD5	8.4 ± 8.3	5.5 ± 4.5	13.7 ± 10.7	** < 0.0001**
GPS				
POD1	1.7 ± 1.2	1.6 ± 1.1	2.0 ± 1.3	**0.002**
POD3	2.9 ± 2.5	2.3 ± 1.6	4.6 ± 3.3	** < 0.0001**
POD5	2.7 ± 3.3	1.6 ± 1.5	4.6 ± 4.7	** < 0.0001**
PCT (ng/mL)				
POD1	0.7 ± 3.1	0.6 ± 2.4	1.1 ± 4.6	0.193
POD3	1.2 ± 5.5	0.9 ± 4.7	1.8 ± 7.4	0.176
POD5	1.1 ± 4.5	0.5 ± 1.8	2.3 ± 7.2	**0.002**
PAR				
POD1	0.2 ± 1.2	0.2 ± 0.8	0.4 ± 1.9	0.142
POD3	0.4 ± 2.0	0.3 ± 1.5	0.7 ± 2.8	0.126
POD5	0.4 ± 2.1	0.2 ± 0.6	1.0 ± 3.5	**0.003**

Bold values indicate statistical significance (*p* <0.05)

*POD* postoperative day,* ALB* albumin,* CRP* C-reactive protein,* GPS* Glasgow Prognostic Score,* LC* lymphocyte count,* NLR* neutrophil–lymphocyte ratio, * NC* neutrophil count,* PAR* procalcitonin to albumin ratio,* PC* platelet count,* PLR* platelet–lymphocyte ratio,* WCC* white cell count

Univariate analysis was carried out by using the Student* t*-test

In summary, NLS, GPS, and CRP presented the higher values at POD1 and POD3 associated with postoperative complications.

The mean value of CRP at POD1 and POD3 for patients with complications were significantly higher compared with patients without complications (POD1 6.9 mg/dl versus 5.7 mg/dl, *p* = 0.006; POD3 14.8 mg/dl versus 7.8 mg/dl, *p* < 0.001).

The mean value of GPS at POD1 and POD3 for patients with complications were significantly higher compared with patients without complications (POD1 2.0 versus 1.6, *p* = 0.002; POD3 4.6 versus 2.3, *p* < 0.001).

The mean value of NLR at POD1 and POD3 for patients with complications were significantly higher compared with patients without complications (POD1 10.6 versus 8.8, *p* = 0.01; POD3 8.0 versus 4.1, *p* < 0.001).

### Cutoff values associated with complications

Since the main objective of this study was to use IM as early predictors of complications, the results of POD5 or POD4 (the day of discharge), whichever came first, despite being more promising, were not taken into consideration for the selection of a prediction cutoff.

For CRP, a cutoff of 12.51 mg/dL on POD3 optimized the sensitivity (54.3%), specificity (80.2%), and accuracy (71.2%) to predict any complications. For GPS, the best predicting cutoff value was 2.12 on POD3 with sensitivity of 78.1%, specificity of 55.8%, and accuracy of 63.6%. Finally, for NLR, a cutoff of 5.26 on POD3 optimized the sensitivity (68.6%), specificity (77.6%), and accuracy (74.6%).

### Negative predictive value

The negative predictive value (NPV) emerged as the most reliable value for predicting the absence of complications.

NLR was the best parameter to identify patients unlikely to experience a postoperative complication on day 3, with a cutoff value of 5.26 and a negative predictive value (NPV) of 83%. Similar results were obtained for GPS and CRP, with cutoff values of 2.12 (NPV of 82.7%) and 12.51 mg/dL (NPV of 76.1%) at POD3, respectively (Table 3). We accurately identified patients unlikely to experience complications as early as postoperative day 3 (POD3).

**Table 3:** ROC curve analyses for selected inflammatory markers (CRP, GPS, and NLR) on POD1, POD3, and POD5

	AUC	Cutoff value	Sensitivity (%)	Specificity (%)	NPV (%)	Accuracy (%)
CRP (mg/dL)						
POD1 (*n* = 397)	0.577	5.18	61.0	53.8	72.1	56.3
POD3 (*n* = 397)	0.727	12.51	54.3	80.2	76.7	71.2
POD5 (*n* = 320)	0.768	93	60.0	84.3	79.8	75.8
GPS						
POD1 (*n* = 382)	0.578	1.17	71.4	42.6	73.7	52.6
POD3 (*n* = 390)	0.720	2.12	78.1	55.8	82.7	63.6
POD5 (*n* = 315)	0.766	2.74	61.9	83.2	80.0	75.5
NLR						
POD1 (*n* = 397)	0.599	10.48	45.3	73.5	72.7	64.1
POD3 (*n* = 397)	0.774	5.26	68.6	77.6	83.0	74.6
POD5 (*n* = 321)	0.753	3.95	66.3	76.5	81.8	73.0

*POD* postoperative day, *AUC* area under the curve, *CRP* C-reactive protein, *GPS* Glasgow Prognostic Score, *NLR* neutrophil–lymphocyte ratio, *NPV* negative predictive value, *ROC curve* receiver-operating characteristic curve

## Discussion

Our study showed that most of the postoperative inflammatory parameters analyzed were significantly higher in patients who developed postoperative complications. After reviewing all the markers’ potential on POD1, POD3, and POD5, we found CRP, GPS, and NLR to be most associated with the development of complications.

Early detection and management of postoperative complications is critical to reduce the morbidity and mortality associated with surgical procedures [15, 16]. Some studies have shown a correlation between time of intervention and severity of septic complications [5, 17]. Therefore, by relying solely on clinical parameters to identify complications, we risk losing valuable time that could be critical for patient outcomes. Clinical signs often appear only after a more advanced stage, missing the crucial window where early and less invasive treatments could be most effective. The current timing of clinical detection of postoperative complications is between a median of 5 and 8 days [5–8].

The present study suggests that routine use of inflammatory markers may allow earlier detection of postoperative complications for patients with colorectal cancer after MIS surgery.

Regarding CRP, a wide variation in cutoff values has been suggested by several studies concerning POD3, with a reported range from 130 to 180 mg/L for the prediction of PC, being the presence of anastomotic leak or infectious complications the most common primary endpoints [9, 14, 18–23]. This variation can be explained because of heterogeneous inclusion criteria within these studies, with most of them accepting multiple indications for elective colorectal surgery, such as interventions for inflammatory bowel disease, and emergency procedures being considered as well.

Furthermore, only specific complications have been analyzed, such as anastomotic leak or inflammatory complications, and at different endpoints. In our study, we include all complications to identify any alterations that may require medical treatment, regardless of their nature.

The results obtained in our analysis suggest that NLR is the best parameter to predict postoperative complications (AUC 0.774) on POD3. The determination of a postoperative cutoff value for NLR was first proposed by Cook et al. (2007) [10], on a prospective cohort study suggesting that NLR ≥ 9.3 on POD1 was associated with an increased risk of complications.

Regarding GPS, Goulart et al. (2018) [24] suggested a cutoff of 4.3 on POD3 for all infectious complications with a NPV of 90.7%, in a prospective cohort with CRC. The higher value compared with our results may be explained owing to the inclusion of only infectious complications, making the test more specific, but at the same time not useful for other noninflammatory complications. Overall, GPS was associated with higher positive predictive values (PPV) for postoperative complications. [25, 26]

In our study, the negative predictive value was the best parameter for identifying patients unlikely to develop postoperative complications. . This information is equally useful, as it enables safe and confident decisions regarding early discharge after MIS surgery for colorectal cancer. Moreover, several studies have already shown that it is possible to securely discharge a patient on POD3 [27–29] or even suggest that a selected group of patients could be discharged as early as the first postoperative day following minimally invasive colorectal surgery [30] . In this study, the development of any postoperative complication is unlikely if the NLR is under a cutoff of 5.26 on POD3 (NPV of 83.0%).

The present study has several limitations that need to be addressed. First, the retrospective nature of the study could introduce biases related to data collection and analysis. The sample size may not be large enough, considering that we only had 116 patients with postoperative complications, limiting the statistical power and extrapolation to all populations undergoing colorectal cancer surgery. Also, as POD5 was analyzed as either day 4 or 5 depending on the discharge date, this inconsistency may introduce variability, which should be considered when interpreting the findings.

## Conclusions

Neutrophil–lymphocyte ratio, C-reactive protein, and Glasgow Prognostic Score on POD3 can predict postoperative complications in patients who undergo minimally invasive surgery for colorectal cancer. In addition, these inflammatory markers showed a high negative predictive value, effectively identifying patients who were unlikely to develop complications, providing important information for safe early discharge.

Patients exceeding the cutoff values identified in our analysis would likely benefit from closer postoperative surveillance and, where appropriate, additional diagnostic tests to promptly identify and address potential complications. As a retrospective, single-center study, we suggest further multicenter prospective studies to validate our findings, its clinical practice significance, and external validation.

## Data Availability

No datasets were generated or analyzed during the current study.
